# Structural Characterization and Optimization of a Miconazole Oral Gel

**DOI:** 10.3390/polym14225011

**Published:** 2022-11-18

**Authors:** Andrada Pintea, Robert-Alexandru Vlad, Paula Antonoaea, Emöke Margit Rédai, Nicoleta Todoran, Enikő-Csilla Barabás, Adriana Ciurba

**Affiliations:** 1 Pharmaceutical Technology and Cosmetology Department, Faculty of Pharmacy, George Emil Palade University of Medicine, Pharmacy, Science and Technology of Targu Mures, 540142 Targu Mures, Romania; 2 Cellular Biology and Microbiology Department, Faculty of Pharmacy, George Emil Palade University of Medicine, Pharmacy, Science and Technology of Targu Mures, 540142 Targu Mures, Romania; 3 Department of Laboratory Medicine, Mures, County Hospital, 540136 Targu Mures, Romania

**Keywords:** miconazole gels, Carbopol, D-optimal drug design, pharmacotechnical evaluation

## Abstract

The development of semisolid formulations, gels in particular, has raised the attention of scientists more and more over the last decades. Because of their biocompatibility, hydrophilic nature, and capacity of absorbing large quantities of water, hydrogels are still one of the most promising pharmaceutical formulations in the pharmaceutical industry. The purpose of this study is to develop an optimal formulation capable of incorporating a water-poorly soluble active ingredient such as miconazole used in the treatment of fungal infections with *Candida albicans* and *Candida parapsilosis*. A D-optimal design was applied to study the relationship between the formulation parameter and the gel characteristics. The independent parameters used in this study were the Carbopol 940 concentration (the polymer used to obtain the gel matrix), the sodium hydroxide amount, and the presence/absence of miconazole. Ten different dependent parameters (Y1–Y10) were evaluated (penetrometry, spreadability, viscosity, and tangential tension at 1 and 11 levels of speed whilst destructuring and during the reorganization of the gel matrix). The consistency of the gels ranged from 23.2 mm (GO2) to 29.6 mm (GM5). The least spreadable gel was GO7 (1384 mm^2^), whilst the gel that presented the best spreadability was GO1 (3525 mm^2^). The viscosity and the tangential stress at the selected levels (1 and 11) varied due to the different compositions of the proposed gels. The gels were also tested for drug content and antifungal activity. All determinations had satisfying results; the drug content was within limits accepted by Ph. Eur. 10 and all formulations containing miconazole exhibited antifungal activity. An optimal formulation with miconazole was attained, consisting of 0.84% Carbopol 940 and 0.32% sodium hydroxide.

## 1. Introduction

Over the last decades, the attention of the scientific world has focused more and more on studying new topical formulations that are physically, chemically, and biologically stable. These pharmaceutical formulations are intended to produce the required therapeutic action at specific targets in the skin with the least side effects possible. One big advantage is that they avoid the hepatic first pass, being easily administrated and transported [1,2]. The time span and intensity of the therapeutic effect depend on the release of the active drug substance and the penetration/diffusion through the stratum corneum and other layers of the skin before performing the desired effect [3]. There are many topical formulations available on the market, however semisolids (e.g., ointments, gels, and creams) are the most commonly used for this purpose [4].

Due to the multiple physicochemical and biological properties of the skin/mucous barrier function, there may be obstacles to drug delivery. The main strategies used to increase the bioavailability are the utilization of nanoparticles, alone or in combination with permeation enhancers or mucoadhesive polymers [5,6,7,8]. The mucous membrane that lines the structures within the oral cavity limits is known as oral mucosa. This is a wet soft tissue membrane that has two layers: the surface stratified squamous epithelium and the deeper lamina propria [9,10,11,12].

Hydrogels are three-dimensional, cross-linked, and supramolecular networks that can absorb large quantities of water. Due to their biocompatibility, hydrophilicity, and therapeutic potential, they are one of the most promising pharmaceutical formulations. Their properties are determined by the cross-linking degree and nature, the tacticity, and the crystallinity of the polymer [13,14,15,16].

In a hydrogel preparation technique, monomers or polymers and a neutralizing agent (such as 10% sodium hydroxide) are usually necessary [14]. Carbopol polymers (CBP) are high-molecular-weighted, cross-linked, and acrylic acid-based polymers. Aqueous dispersions of CBP polymers have an approximate pH range of 2.8 to 3.2, depending on the concentration of the polymer. They can absorb large quantities of water, become hydrated, and swell. Their hydrophilic nature, cross-linked structure, and insolubility in water make them good candidates for controlled-release drug delivery systems. Depending on the cross-linking degree and the manufacturing process, there are various grades of CBP polymers available on the market, each of them having its significance in pharmaceutical dosage forms. CBP 940 is a gelling agent used to increase the viscosity of the formulation while also being able to create bonds with the mucous membrane, resulting in strong bioadhesion. In its powder form, the molecules of the polymer are curled tightly, limiting its thickening capacity. When absorbing water, the molecules start to uncoil, resulting in increased viscosity. To achieve maximum performance of the polymer, the molecule must be completely uncoiled. This can be realized by neutralizing the polymer with a suitable alkaline ingredient (e.g., sodium hydroxide, potassium hydroxide, or triethanolamine), Figure S1 [17,18,19,20,21]. These types of gels can be used to obtain dispersions with substances that can be included in different classes of the Biopharmaceutics Classification System (BCS) (e.g., benzoic acid, boric acid 10%, benzocaine 1%, zinc oxide 20%, sulfathiazole 5%) [22].

Miconazole (MIC, Figure S2) is a white or pale cream, odorless, crystalline, or microcrystalline powder that is insoluble in water and freely soluble in acetone. It is a first-generation synthetic imidazole that exerts a broad spectrum against dermatophytes (e.g., *Trichophyton mentagrophytes*, *Epidermophyton floccosum*), yeasts (e.g., *Candida* spp.), as well as Gram-positive bacteria (e.g., *Staphylococcus aureus*, *Streptococcus faecalis*) [23,24,25,26]. MIC inhibits the cytochrome P450 complex, including the 14 α-demethylase enzyme required for ergosterol synthesis in the fungal cell membrane. In addition, toxic sterols accumulate in the cell and the synthesis of triglycerides and phospholipids is altered [27,28]. Because of its poor oral absorption and rapid clearance the main administration route is the topical one [22,29]. Furthermore, systemic use is limited because of its many side effects (e.g., pruritus, anemia, nausea, thrombocytosis, and central nervous system effects) [30].

MIC is usually incorporated into semisolid formulations intended for application in the oral cavity to treat oral candidiasis [31]. Due to its limited solubility in water, permeation through the biological membranes is low and, as a result, its pharmacological action can be compromised. Oral mucosa offers several advantages for drug delivery, such as high vascularization, the avoidance of first-pass metabolism, and low enzymatic activity, which could improve drug bioavailability and patient compliance [32]. The drug is dispersed in a mucoadhesive polymer that swells in the presence of water and exhibits bioadhesive properties, making it adhesive to the skin for a prolonged time and releasing it gradually, this way offering a slow diffusional path length and reducing concentration fluctuations [33,34].

Recently, the concept of Quality by Design (QbD) has caught the attention of many scientists in the pharmaceutical industry. The classic formulation development technique is both empirical and challenging, while the quality is not guaranteed. This process aims to design the quality of the product, not to test it. The QbD process is a scientific, systematic, and risk-based method for developing, formulating, and controlling processes, to achieve the desired quality of the product. This evaluation is done by identifying and studying the interaction between independent and dependent variables and how they affect product performance; an approach that results in fewer experiments that are time- and cost-efficient. The main elements of QbD are defining desired product profiles, designing product and manufacturing processes, and identifying critical quality parameters and variables [35,36,37,38,39,40]. For developing mucoadhesive gels containing MIC, a D-optimal design with three variables was used. Using the software MODDE^®^ 13.1, a final optimal formulation was developed.

Throughout the years, the incidence of fungal infections has rapidly increased due to the susceptibility of humans to infections [41,42]. Considering the lack of oral gel formulations containing MIC in the Romanian pharmaceutical market, the purpose of this study is to develop MIC gels that treat oral thrush (aphthae) and to analyze their properties in order to achieve an optimal formulation.

## 2. Materials and Methods

### 2.1. Gel Preparation

Two series of gel formulations were prepared, as presented in Table 1. Blank gels were formulated by dissolving citric acid (taste masker, salivation stimulator), sucralose (sweetener), and caramel flavor in a preservative solution, under continuous stirring. The solution was heated at 35 °C to facilitate a complete dissolution. After the mixture was cooled, the alcohol (co-solvent) was added in drops. The stated quantity of CBP (gelling agent) was sprinkled evenly on the surface of the solution and was left in repose for 1 h. Afterward, while stirring, the sodium hydroxide solution was added to neutralize and adjust the pH of the solution, followed by the required amount of glycerol. The second series of 7 gels were prepared by following the same process. In addition, MIC was incorporated by dispersing it in a small quantity of blank gel, while mixing continuously to obtain a suspension gel. Gradually, the whole amount of blank gel was added. 

### 2.2. Developing the Optimal Formulation Using a D-Optimal Design

Using the MODDE 13.1 software, the interaction between the independent variables (Table 2) and the dependent variables (Table 3) was studied using a D-optimal design to determine the optimal formulation of the gel. The statistical parameters used to evaluate the proposed model are the coefficient of determination R^2^, which indicates the variance in the response variable as explained by the model, and Q^2^, which indicates the variation of the response as predicted by the model, model validity, and reproducibility. Based on the statistical data, the software develops an equation from which the influence of the independent variables on the response can be evaluated [43,44,45]. The goal is to optimize the independent variables to achieve the desired values of the dependent parameters [46]. Fourteen gels were prepared (seven with MIC and seven without MIC), respecting a D-optimal design using the previously mentioned software. The following dependent parameters were evaluated: consistency, spreadability, tangential stress, and viscosity while destructuring and reorganizing at speed levels 1 and 11.

#### 2.2.1. Consistency

To measure the consistency, a manual penetrometer (Koehler K19500, Koehler Instrument Co., Inc., New York, NY, USA) was used; it has a conic metal part attached to a bar and, by free falling, settles on the center of the surface of the gel. An amount of gel was put into a plastic box and the penetration from the surface of the gel to the peak of the cone was measured. Weights ranging from 5–40 g were added to the cone, noticing the penetration depth after every measurement.

#### 2.2.2. Spreadability

The spreadability determination was carried out using the Del Pozo Ojeda-Suñé Arbussá extensometer by placing 1 g of gel between two glass plates and adding weights between 100 and 500 g at an interval of 1 min. The diameter occupied by the gel was noted after each measurement.

#### 2.2.3. Rheological Study

The rheological properties were determined using a Rheotest^®^ RV viscometer (RHEOTEST Medingen GmbH, Ottendorf-Okrilla, Germany) at 21 ± 2 °C, by completing 12 speed levels. Firstly, the speed levels increased from 1 to 12, destructuring the gel-matrix, and, after reaching the highest level of speed (12), the gel was restructured by decreasing the speed gradually from 12 to 1 using the H/H cylinders. 

### 2.3. Drug Content

To determine the drug content, 1 g of gel from each formulation was dissolved into 50 mL of methanol and extracted from the polymeric matrix using the MS-H280-Pro DLAB agitator (DLAB, La Mirada, CA, USA) at 800 rotations/min. The samples were stirred for 30 min. A series of dilutions were applied to enclose the amount of MIC in the concentration used to obtain the calibration curve. The absorbance of the solution was determined using the UV-1800 Shimadzu Spectrophotometer (Mettler Toledo, Columbus, OH, USA) at 206.2 nm against a blank solution.

### 2.4. Antifungal Activity

To cultivate the *Candida* ssp., the Sabouraud agar medium (SAM) was used. The cultivation of the SAM took 48 h. Gels containing miconazole nitrate (GM1-GM7) and blank gel GO5 were used to study the antifungal activity against *Candida albicans* and *Candida parapsilosis*. Spores from fungus cultures were mixed with the agar plates and small amounts of gels were brought on. The plates were incubated at 37 °C for 24 h and were examined for the inhibition zone diameter.

## 3. Results and Discussion

### 3.1. Appearance

The gels were verified in terms of clarity, color, and transparency. The blank series (GO1-GO7) consisted of transparent and thick gels shown in Figure S3a. The seven gels containing the active drug (GM1-GM7) presented opaque, white, and thick characteristics as illustrated in Figure S3b.

### 3.2. Evaluation of the Dependent Parameters Using a D-Optimal Design

The results regarding the evaluated dependent parameters are included in Table 4.

**Figure 1:**
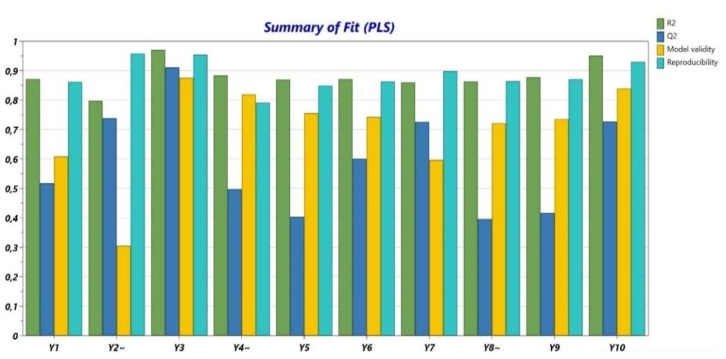
Summary of fit (R^2^, Q^2^, model validity, and reproducibility).

The obtained data from the performed determinations were analyzed from a statistical point of view. R^2^ (Figure 1) presented values higher than 0.8 for all variables and Q^2^ (Figure 1) showed values between 0.4 and 0.9. The validity was higher than 0.25 for all variables, with values close to 0.9 for the majority of the experiments. Usually, when the validity values are under 0.25, there is a high risk of a lack of fit. The values for reproducibility were over 0.8 for all determinations. Additionally, the *p*-ANOVA and the lack of fit were evaluated (Table 5). The non-significant terms were eliminated.

The ANOVA test showed that the selected independent factors had an increased impact on the responses and the lack of fit for all 10 variables was excluded (Table 5).

**Figure 2:**
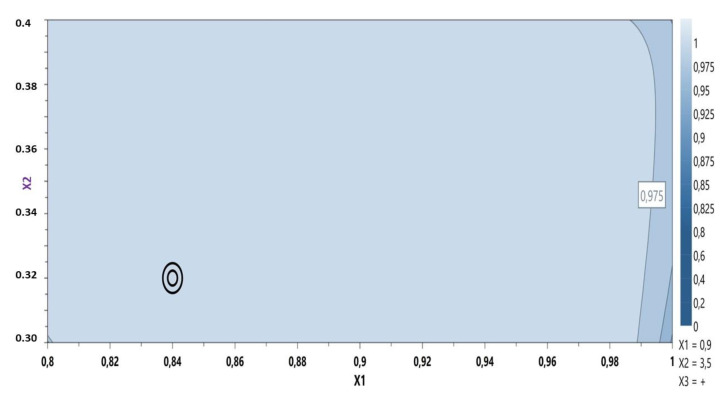
Desirability plot: MIC gel optimization.

Based on these results the model was considered fitted and the optimal formulation was proposed using the desirability plot: CBP 0.84%, sodium hydroxide 0.32% containing the active ingredient (Figure 2).

#### 3.2.1. Consistency

**Figure 3:**
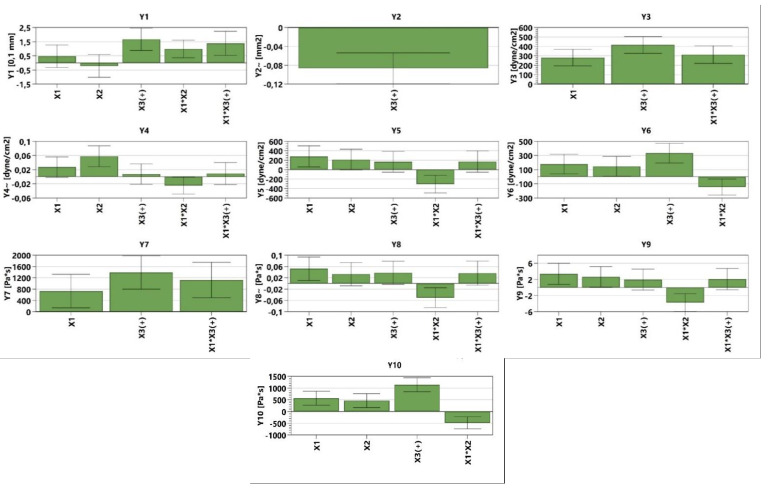
The influence of the independent variables on the dependent variables.

**Figure 4:**
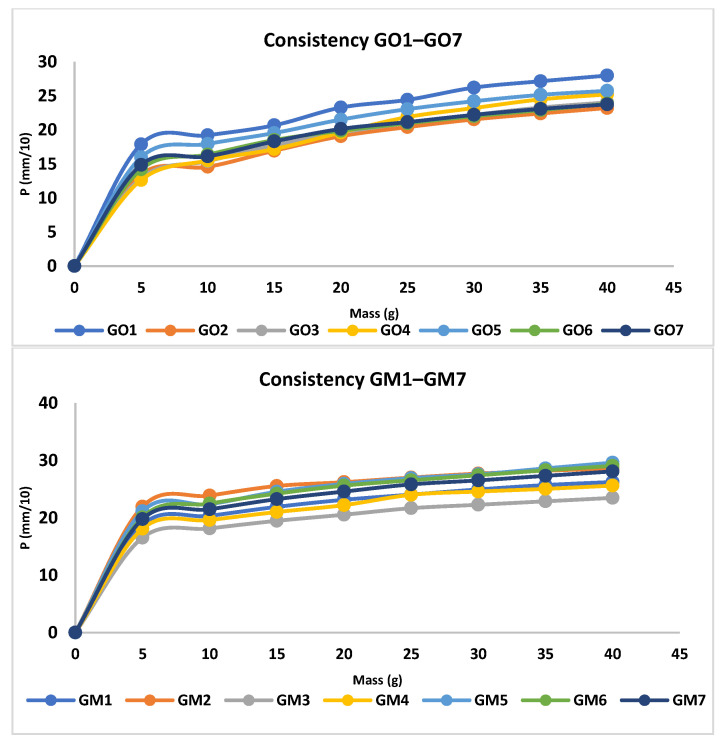
Consistency profiles for MIC and blank gels.

The analysis of the consistency of the gels shows that gels containing MIC have been more easily infiltrated compared with the blank series of gels. The consistency increases directly proportional to the weight applied. In addition, according to the design used, the factors that are significantly influencing the consistency are X3 and the interaction between X1×X2 and X1×X3; all of them conducting in an increased penetration (Figure 3, Table S1, and Equation (S1)). X1 and X2 are influencing the consistency differently; X1 increases the penetration capability, whilst X2 decreases the penetration. The last two factors have a reduced influence on consistency compared with X3 and the interaction between X1×X2 and X1×X3. The gel with the highest penetration capacity is GM5 (29 mm), whilst the lowest penetration capacity is registered in the case of GO2 (23.2 mm) (Figure 4 and Table 4).

#### 3.2.2. Spreadability

**Figure 5:**
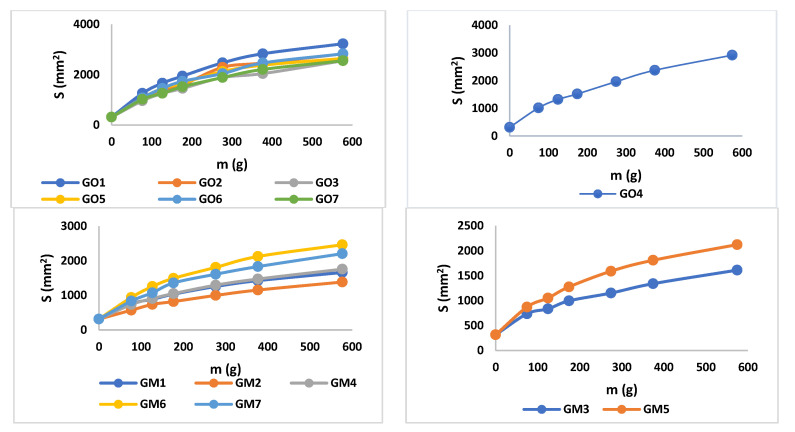
Spreadability profiles for blank and MIC gels.

The values of spreadability demonstrate that the blank series of gels (GO1–GO7) are more easily spreadable than GM1–GM7, while applying the same weight. By increasing the weight, the evaluated gels spread progressively; a behavior mentioned also by Muț et al., where fluconazole hydrogels were developed and evaluated [47]. The spreadability profiles can be retrieved in Figure 5 and the final values obtained during the experiment can be observed in Table 4. The highest spreadability value is 32,525.43 mm^2^ (GO1), while the lowest is 1384.74 mm^2^ (GM2). Studies have shown that the concentration of the gelling agent is inversely proportional to the spreadability capacity [48]. The main parameter that affects this parameter is the presence of MIC (X3) (Figure 3, Table S1 and Equation (S2)), which conducts to a decreased value regarding the spreadability.

#### 3.2.3. Rheology Study

**Figure 6:**
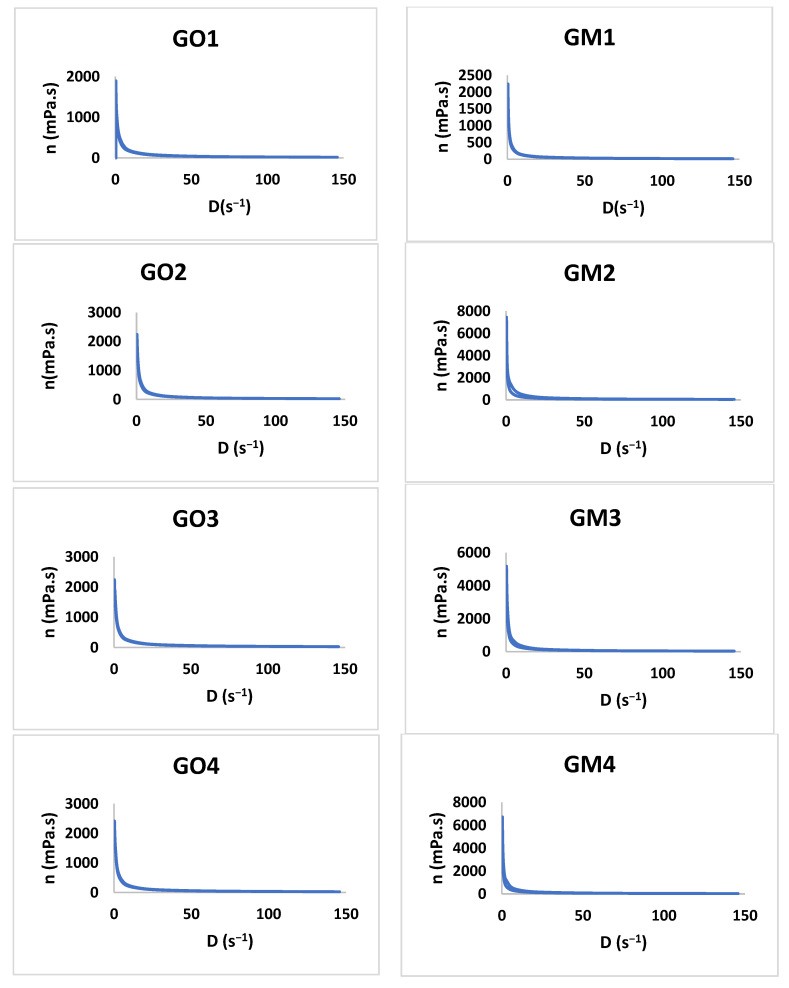

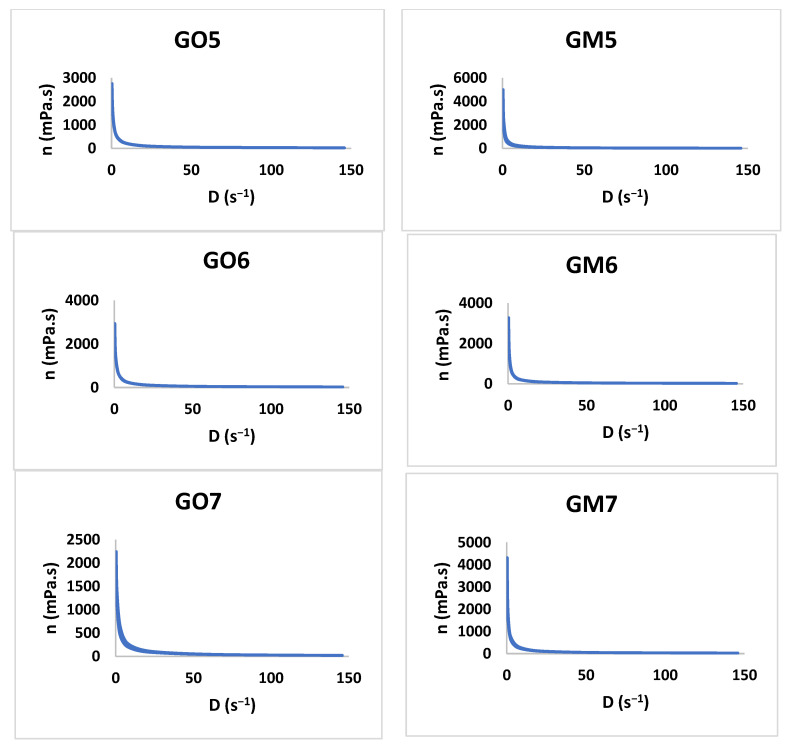
The viscosity profiles for GO1-GO7 and GM1 and GM7.

The study shows that all formulations have a pseudoplastic flow (Figure 6). The literature shows that increasing the concentration of the polymer and the neutralizer implies an increased viscosity [48,49,50,51]. By increasing the tangential stress, the flow rate increases, and the viscosity decreases (Table 4). Additionally, the initial viscosity can be compared for all the gels developed considering the values obtained for the Y7 independent variable. For six out of seven gel formulations (1–6), the presence of MIC in the polymeric matrix conducts to an increased viscosity value in comparison with the blank gels.

The tangential stress at the first level is influenced positively by X1, X3, and the interaction between X1×X3, increasing the values of the tangential stress. In the case of Y4 (Figure 3, Table S1 and Equation (S4)), it can be noticed that X1, X2, X3, and the interaction X1×X3 produce higher tangential stress. In the case of the latter, the influence is lower compared with the first three parameters. The interaction between X1×X2 conducts to lower tangential stress. The interaction between X1×X2 shows the same behavior in the case of Y5 (Figure 3, Table S1 and Equation (S5)) and Y6 (Figure 3, Table S1 and Equation (S6)) (decreases tangential stress). The dependent parameters X1, X2, and X3 conduct to an increased value regarding the tangential stress, whilst in the case of Y5, the interaction of X1×X3 exhibits the same behavior. The viscosity at different levels is influenced by the following dependent parameters: X1, X3, and X1×X3, all three of them increasing the viscosity for the independent parameters Y7–Y9 (Figure 3, Table S1, Equations (S7)–(S9)). The Y8–Y10 (Figure 3, Table S1 and Equations (S8)–(S10)) parameters are also influenced by the X2 (increased viscosity) and the interaction between X1×X2 that decreases the viscosity. It can be noticed that the interaction between X1×X2 decreases both the tangential stress and the viscosity while destructuring the gel at level 11 and while the system is reorganizing at both level 11 and level 1 (Figure 3).

The rotational viscometer is extensively used to determine the rheological properties of semisolid formulations (gels, ophthalmic gels, creams, and ointments) [52,53,54]. In this manner, it has to be considered that the determinations were made at room temperature. In the future, an evaluation at 37 ± 1 °C might be necessary because the temperature might be an important variable that could influence the gel’s rheological properties [54].

### 3.3. Drug Content

The gels containing MIC were analyzed, and all formulations GM1–GM7 were within the limit accepted by the *European Pharmacopoeia*, 10th Edition, Ph. Eur. 10, as shown in Table 6.

### 3.4. Antifungal Activity

**Figure 7:**
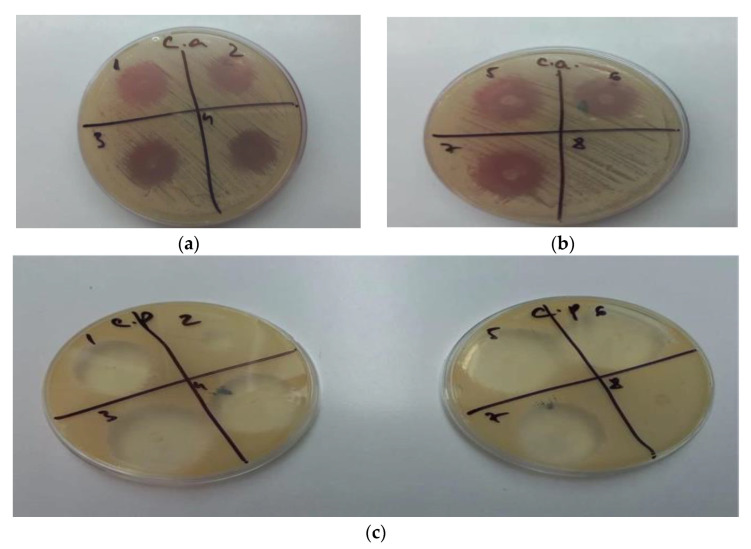
(**a**) GM1–GM4 (1–4) inhibition of *Candida albicans*; (**b**) GM5–GM7 (5–7), GO5 (8) inhibition of *Candida albicans;* (**c**) GM1–GM7 (1–7), GO5 (8) inhibition of *Candida parapsilosis*.

The study revealed that gels GM1–GM7 (noted as 1–7), have inhibited the growth of *Candida albicans* and *Candida parapsilosis,* while the gel used as a blank, GO5, (noted in Figure 7b,c as 8) showed no antifungal activity. In the case of GM2, the antifungal effect was lower in the case of *Candida parapsilosis* compared with the other formulation. Other than oral gels, MIC can also be incorporated into tissue conditioners for the treatment of denture stomatitis. Moreover, other antifungal agents were reported to be integrated into tissue conditioners, e.g., nystatin, fluconazole, and amphotericin B [55]. In the study conducted by Rodnai et al., where the MIC was incorporated in visco-gel, the antimycotic effect of five different concentrations (ranging from 5% to 25%–*v*/*v*%) was evaluated, noticing that an increased MIC concentration conducts to an increased inhibition zone around the MIC discs [56]. The 2% (*w*/*w*) MIC gels developed in this study provide the necessary antimycotic activity, as shown in Figure 7a–c.

### 3.5. Optimization of the MIC Gel Formulation

The optimized MIC gel formulation consisted of 0.84% CBP 940 and 0.32% NaOH; all the other excipients being maintained in the same amount. For a better comparison, a blank gel with the same composition was developed. The experimental data for the dependent variables proposed can be found in Table 7. Considering the results obtained, the prediction is fitted for 9 out of 10 answers and the only result belonging to the optimized gel that has not fit the prediction range is Y3, where the tangential stress at level 1 was smaller than the predicted minimum (min) value proposed by the software used.

**Figure 8:**
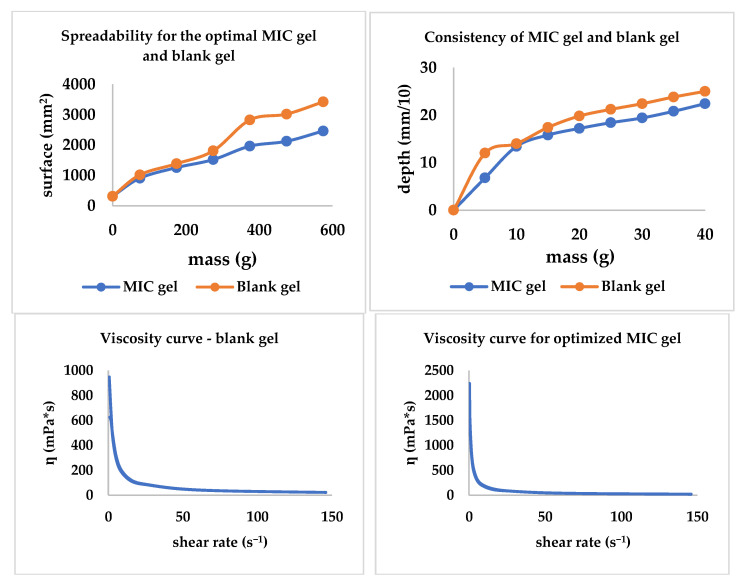
The consistency, spreadability, and viscosity curves for the optimized gel and the blank gel with the same composition.

The spreadability, consistency, and viscosity curves were traced; all three are included in Figure 8. The MIC gel and the blank gel presented comparable behavior regarding spreadability and consistency. Additionally, through this experiment, the influence of the presence/absence of MIC is highlighted, with the MIC decreasing both spreadability and consistency. Regarding the viscosity, MIC presence tends to increase the viscosity behavior revealed in Figure 8.

The spreadability, consistency, and rheological characteristics of the MIC gel contribute to the ease of administration (application) [57,58,59,60]. Furthermore, an easily spreadable gel might be preferred by patients suffering from aphthae.

#### 3.5.1. Uniformity of Content

The uniformity of content revealed that the MIC concentration for the optimized gel is in the range admitted by the in-force Ph. Eur. 2 ± 0.3%, with a concentration of 2.05 ± 0.05%.

#### 3.5.2. Antifungal Activity of the Optimized Gel

**Figure 9:**
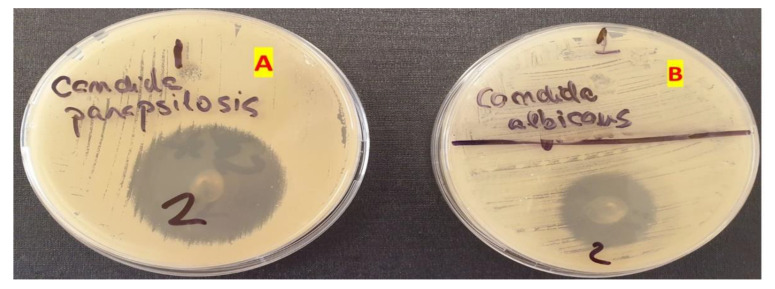
The inhibition of *Candida parapsilosis* (**A**) and *Candida albicans* (**B**) using the blank gel (1) and the optimized MIC gel (2).

The antifungal activity of the optimized gel for both *Candida albicans* and *Candida parapsilosis* can be retrieved in Figure 9. For a better comparison, the MIC gel antifungal activity was compared with the one belonging to the blank gel. The blank gel showed no activity on the two fungi, whilst the optimized gel inhibited the development of both *Candida albicans* and *Candida parapsilosis*.

## 4. Conclusions

The presence of MIC can conduct to a weakened gel matrix, decreasing the consistency fact indicated through the penetrometry study, where the gels without MIC (GO1–GO7) presented a lower penetration capacity compared with those with MIC (GM1–GM7). This being taken into consideration, it can be concluded that, in the case of the blank gels, the force opposing the penetration is higher compared with MIC gels. In the case of the optimized blank and MIC gel, the results regarding consistency are comparable. The active pharmaceutical ingredient (API) also exhibited a negative effect regarding the Y2 (spreadability) decreasing in the case of the MIC gels compared with the blank ones.

At a lower level of speed during the gel matrix disorganization, the tangential stress and viscosity were not influenced by the concentration of diluted sodium hydroxide, whilst, when the speed increased and during the reorganizing of the gel matrix, this factor conducted for both tangential stress and viscosity to increase. As expected, the CBP concentration conducted to an increased tangential stress and viscosity. In addition, the API increased the tangential stress in the case of Y3, Y5, and Y6 and viscosity for all four independent parameters coded as Y7–Y10.

Antimycotic oral gels were developed and optimized using a D-optimal design. The results obtained in this study showed that the gels present good spreadability and consistency, appropriate rheological behavior, drug content within the admitted limits, and antifungal activity. Moreover, it has been demonstrated that the active drug plays a significant role in developing the desired product, influencing the selected independent parameters. An optimized formulation was developed, evaluated, and compared with the predicted results, with the achieved results ranging between the min and the max predicted values.

## Figures and Tables

**Table 1:** Composition of the two studied series of gels.

Ingredient% (w/w)	Gel	G1	G2	G3	G4	G5	G6	G7
Miconazole nitrate ^1^/Gels Series	**M**	2	2	2	2	2	2	2
	**O**	-	-	-	-	-	-	-
CBP 940 ^2^		0.8	1	0.8	1	0.9	0.9	0.9
Sodium hydroxide ^3^		0.3	0.3	0.4	0.4	0.35	0.35	0.35
Glycerol ^4^		3	3	3	3	3	3	3
Sucralose ^5^		0.05	0.05	0.05	0.05	0.05	0.05	0.05
Citric acid ^6^		0.1	0.1	0.1	0.1	0.1	0.1	0.1
Caramel ^7^		0.2	0.2	0.2	0.2	0.2	0.2	0.2
Alcohol ^8^		0.2	0.2	0.2	0.2	0.2	0.2	0.2
Preservative solution ^9^		ad 100	ad 100	ad 100	ad 100	ad 100	ad 100	ad 100

1. GO—blank gels series (1–7), 2. GM—miconazole nitrate gels series (1–7). ^1^ Miconazole nitrate (Fengchen Group Co., Qingdao, China); ^2^ CBP 940 (3V Sigma SPA, Bergamo, Italy); ^3^ sodium hydroxide (Merck, Darmstadt, Germany); ^4^ glycerol (Fengchen Group Co., Qingdao, China); ^5^ sucralose (My protein, Manchester, UK); ^6^ citric acid (Chemical Company, Iasi, Romania); ^7^ caramel (prepared in the Pharmaceutical Technology and Cosmetology Laboratory); ^8^ alcohol (Chimreactiv SRL, Bucharest, Romania); ^9^ preservative solution (prepared in the Pharmaceutical Technology and Cosmetology Laboratory using Nipagin-Fagron, Trikala, Greece and Nipasol-Fagron, Trikala, Greece).

**Table 2:** Independent parameters.

Independent Factors	Code	Level		
CBP concentration	X1	−1	0	+1
		0.8	0.9	1
Sodium hydroxide amount	X2	−1	0	+1
		0.3%	0.35%	0.4%
MIC content	X3	+(gels with MIC)	−(gels without MIC)	

**Table 3:** The dependent parameters evaluated.

Dependent Parameter Name	Code	Measuring Unit
Consistency	Y1	mm
Spreadability	Y2	mm^2^
Destructuring tangential stress, level 1	Y3	dyne/cm^2^
Destructuring tangential stress, level 11	Y4	dyne/cm^2^
Reorganization tangential stress, level 11	Y5	dyne/cm^2^
Reorganization tangential stress, level 1	Y6	dyne/cm^2^
Destructuring viscosity, level 1	Y7	Pa × s
Destructuring viscosity, level 11	Y8	Pa × s
>Reorganization viscosity, level 11	Y9	Pa × s
Reorganization viscosity, level 1	Y10	Pa × s

**Table 4:** The dependent parameters (Y1–Y10) for the 14 gel formulations.

Code	Run Order	Y1	Y2	Y3	Y4	Y5	Y6	Y7	Y8	Y9	Y10
GO1	1	27.96	3525	625.9	2333	2276	740	1897	29	28	1897
GO2	2	23.20	2826	739	3072	3015	739	2241	38	37	2241
GO3	3	24.03	2550	769	3414	3243	740	1724	42	40	2241
GO4	4	25.20	2920	797	3357	3186	796	1724	41	39	2413
GO5	5	25.73	2640	910	3357	3015	796	2758	41	37	2413
GO6	6	26.23	1661	739.7	2105	1878	740	2241	26	23	2241
GO7	7	28.56	1384	2457	5692	4269	1747	7445	70	52	5294
GM1	8	23.46	1610	1707	3926	3584	1593	5172	48	44	4827
GM2	9	25.60	1756	2219	3983	3414	1536	6724	49	42	4655
GM3	10	23.83	2826	967	3129	3129	796	2931	38	38	2413
GM4	11	23.73	2550	739	2788	2674	569	2241	34	33	1724
GM5	12	29.60	2122	1650	2901	2560	1194	5000	35	31	3620
GM6	13	29.03	2461	1081	2560	2503	853	3276	31	30	2586
GM7	14	28.52	2205	1422	3186	3129	796	4310	39	38	2413

Y1—consistency; Y2—spreadability; Y3—destructuring tangential stress, level 1; Y4—destructuring tangential stress, level 11; Y5—reorganization tangential stress, level 11; Y6—reorganization tangential stress. level 1; Y7—destructuring viscosity, level 1; Y8—destructuring viscosity, level 11; Y9—reorganization viscosity, level 11; Y10—reorganization viscosity, level 1.

**Table 5:** Quality of fit (R^2^ adjusted and RSD) and the ANOVA test.

Code	Summary of Fit		ANOVA Test	
	R^2^ Adj	RSD	*p*-Value	Lack of Fit
Y1	0.78	1.114	0.005	0.210
Y2	0.78	0.045	<0.001	0.062
Y3	0.97	126.3	<0.001	0.609
Y4	0.79	0.037	0.009	0.486
Y5	0.76	297.8	0.012	0.377
Y6	0.81	208.9	0.001	0.358
Y7	0.81	896.8	<0.001	0.199
Y8	0.75	0.055	0.014	0.329
Y9	0.78	3.480	0.010	0.348
Y10	0.92	417.8	<0.001	0.527

Y1—consistency; Y2—spreadability; Y3—destructuring tangential stress, level 1; Y4—destructuring tangential stress, level 11; Y5—reorganization tangential stress, level 11; Y6—reorganization tangential stress, level 1; Y7—destructuring viscosity, level 1; Y8—destructuring viscosity, level 11; Y9—reorganization viscosity, level 11; Y10—reorganization viscosity, level 1.

**Table 6:** Drug content GM1–GM7.

Code	Drug Content (% w/w) ± SD
GM1	2.148 ± 0.0290
GM2	2.207 ± 0.0127
GM3	1.743 ± 0.0219
GM4	2.284 ± 0.0066
GM5	2.293 ± 0.0071
GM6	1.896 ± 0.0132
GM7	2.212 ± 0.0112

**Table 7:** Experimental data vs. predicted minimum and maximum values.

Name	Predicted Min	Predicted Max	Experimental Data	Fitting Prediction
Y1	22.38	32.47	22.4	Yes
Y2	1855.13	2714.78	2462	Yes
Y3	735.51	2337.64	569	<Predicted min
Y4	2190.17	3803.76	2788	Yes
Y5	2030.53	3981.38	2731	Yes
Y6	17.22	1533.82	739	Yes
Y7	1613.38	6913.43	1724	Yes
Y8	26.73	60.9042	34.42	Yes
Y9	24.94	48.6021	34	Yes
Y10	52.21	4905.9	2242	Yes

## Supplementary Materials

The following supporting information can be downloaded at: https://www.mdpi.com/article/10.3390/polym14225011/s1, Figure S1. Carbopol dispersion in water, Figure S2. Chemical structure of MIC, Figure S3. (a) Blank gel; (b) Gel containing MIC, Table S1. The extended list of coefficients of tablets’ dependent variables, Equations (S1)–(S10).
