# Supplementary material for: Structural Characterization and Optimization of a Miconazole Oral Gel
Source: Polymers (Basel). 2022 Nov 18;14(22):5011. doi: 10.3390/polym14225011 (PMC9692734; doi:10.3390/polym14225011)
Supplement: Supplementary file 1 [file polymers-14-05011-s001.zip › polymers-2038266-supplementary.pdf]

polymers

Article

Structural Characterization and Optimization of a Miconazole oral Gel

Andrada Pintea [1, †], Robert-Alexandru Vlad [1, †], Paula Antonoaea[1, *], Rédai Emőke Margit [1], Todoran Nicoleta [1], Barabás Enikő [2,3], and Adriana Ciurba [1]

Supplementary Materials Section

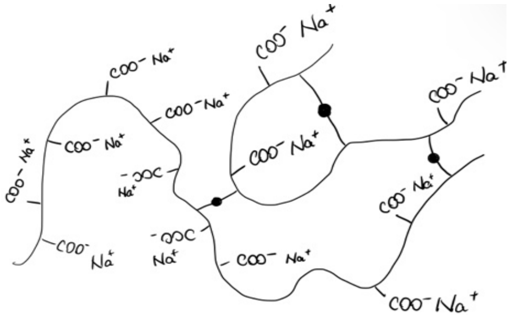

Figure S1. Carbopol dispersion in water

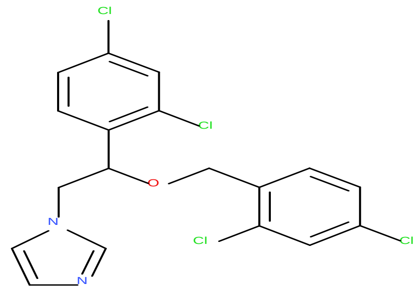

Figure S2. Chemical structure of MIC

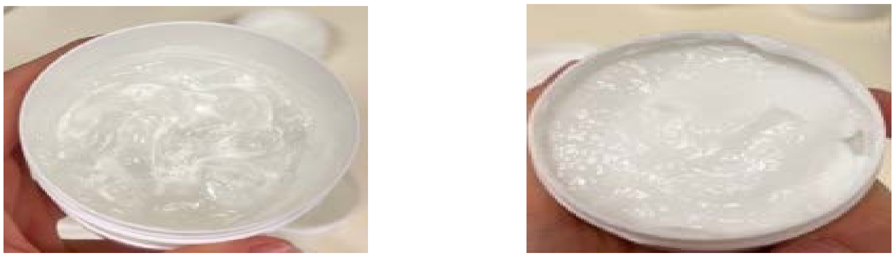

(a) **(b)**

Figure S3. (**a**) Blank gel; (**b**) Gel containing MIC

polymers

MDPI

Table S1. The extended list of coefficients of tablets' dependent variables

Terms	Variables									
	Y1	Y2	Y3	Y4	Y5	Y6	Y7	Y8	Y9	Y10
**Intercept**	26.37	3.36	1132.33	3.49	3067.64	930.65	3173.5	1.6	37.62	2840.17
**X1**	0.47	-	281.08	0.027	279.38	178.16	729.09	0.052	3.36	564.34
**X2**	-0.21	-	-	0.057	212.47	146.87	-	0.033	2.61	464.78
**X3**	1.67	-0.08	415.76	0.007	165.48	333.87	1387.79	0.037	1.96	1136.29
**X1*X2**	0.99	-	-	-0.024	-305.81	-145.58	-	-0.05	-3.73	-481.27
**X1*X3**	1.37	-	312.6	0.009	171.14	-	1120.95	0.036	2.07	
**X2*X3**	-	-	-		-	-	-	-	-	

Equations:

$$Y1=26.37+0,47*X1-0.21*X2+1.67*X3+0,99*X1*X2+1.37*X1*X3 \quad (S1)$$

$$Y2=3.36-0.08*X3 \quad (S2)$$

$$Y3=1132.33+281.08*X1+415.76*X3+312.6*X1*X3 \quad (S3)$$

$$Y4=3.49+0.027*X1+0.057*X2+0.007*X3-0.024*X1*X2+0.009*X1*X3 \quad (S4)$$

$$Y5=3067.64+279.38*X1+212.47*XX2+165.48*X3-305.81*X1*X2+171.14X1*X3 \quad (S5)$$

$$Y6=930.65+178.16*X1+146.87*X2+333,87*X3-145.58*X1*X2 \quad (S6)$$

$$Y7=3173.5+729.09+1387.79*X3+1120.95*X1*X3 \quad (S7)$$

$$Y8=1.6+0.052*X1+0.033*X2+0.037*X3-0.05*X1*X2+0.036*X1*X3 \quad (S8)$$

$$Y9=37.62+3.36*X1+2.61*X2+1.96*X3-3.73*X1*X2+2.07*X1*X3 \quad (S9)$$

$$Y10=2840.17+564.34*X1+464.78*X2+1136.29*X3-481.27*X1*X2 \quad (S10)$$
